# Identification of Quantitative Trait Loci and Analysis of Novel Candidate Genes for Resistance to False Smut of Rice Based on SSR Molecular Markers

**DOI:** 10.3390/biom15020186

**Published:** 2025-01-28

**Authors:** Rongtao Fu, Liyu Zhao, Cheng Chen, Jian Wang, Yu Chen, Daihua Lu

**Affiliations:** 1 Institute of Plant Protection, Sichuan Academy of Agricultural Science, Chengdu 610066, China; furongtao66@scsaas.cn (R.F.);; 2 Key Laboratory of Integrated Pest Management on Crops in Southwest, Ministry of Agriculture and Rural Affairs, Chengdu 610066, China

**Keywords:** rice false smut, *Ustilaginoidea virens*, quantitative trait loci, SSR marker, resistance-related protein

## Abstract

Rice false smut (RFS), an emerging disease caused by the fungus *Ustilaginoidea virens* (Cooke), reduces rice grain yield and quality in rice-planting regions worldwide. The identification of the genes or quantitative trait loci (QTLs) associated with RFS resistance is vital to resistance breeding and the mitigation of RFS damage. In this study, RFS resistance QTLs were located in the resistant variety IR77298-14-1-2::IRGC117374-1. A total of 4 RFS resistance QTLs were detected on rice chromosomes 1, 3, 5, and 12 in the F_2_ and F_4_ mapping populations using 119 polymorphic simple sequence repeat (SSR) molecular markers. Of these QTLs, *qRFS3.01* and *qRFS12.01-1* were repeatedly detected in both populations. Interestingly, QTL *qRFS3.01* on chromosome 3 is a novel resistance locus that exhibited the largest phenotypic effect. These results suggest that SSR markers linked to *qRFS3.01* are valuable for marker-assisted breeding for RFS resistance in rice. The prediction of putative candidate genes within *qRFS3.01* revealed three resistance-related proteins containing an F-box domain, Myb-like DNA-binding domain, and kinase protein. In summary, our findings provide new QTLs/genes for resistance to RFS and will promote rice disease resistance through molecular-marker-assisted breeding.

## 1. Introduction

Rice false smut (RFS) is an emerging fungal disease of rice caused by the ascomycete fungus *Ustilaginoidea virens* (Cooke) Takahashi. This pathogenic fungus mainly infects young rice spikelets and transforms the infected spikelet into false smut balls composed of mycelium and chlamydospores [1]. In recent years, due to the promotion and large-scale planting of high-yield hybrid rice, the excessive use of nitrogen fertilizer has created favorable conditions for the occurrence and prevalence of RFS [2]. In China, the occurrence range of RFS has expanded, and the incidence degree is becoming increasingly serious. The yield loss in severely affected fields can reach more than 50% or even result in no harvest. RFS can not only reduce rice yield and quality but also produce mycotoxins harmful to humans and livestock [3,4].

To date, no rice varieties have been found that are completely immune to *U. virens*, and pathogen resistance varies greatly among rice varieties [5]. Therefore, RFS control is mainly dependent on fungicides at present. RFS symptoms are visible only after flowering, and the use of fungicides for disease control at this stage does not protect the rice plant [6]. Fungicide use may pose risks to the environment and human health, and excessive fungicide use increases the risk of the emergence of resistant *U. virens* strains [7]. However, practice has demonstrated that the identification of resistance genes, the screening of molecular markers closely linked to resistance genes, and the selection of resistant varieties using molecular markers are effective ways to improve the resistance in rice varieties.

RFS resistance in rice is controlled by quantitative trait loci (QTLs) [8,9,10]. More than 40 QTLs conferring RFS resistance have been identified to date. Li et al. [11] identified five QTLs using a recombinant inbred line (RIL) population derived from a cross between resistant variety IR28 and susceptible variety Daguandao. Andargie et al. [9] identified two QTLs conferring RFS resistance on chromosome 5 in IR28 using an F_2_ population derived from a cross between rice cultivars IR28 and HXZ. Han et al. [12] detected five RFS resistance QTLs on chromosomes 2, 4, 8, and 11 in a RIL population derived from a cross between resistant rice landrace MR183-2 and highly susceptible line 08R2394. Hiremath et al. [13] identified the potential donors and QTLs for resistance to false smut in susceptible checks GSR123 and PR116. Qiu et al. [14] fine-mapped one QTL on chromosome 1 using an F_2_ population from a cross between resistant variety Nanjing11 and susceptible variety CG3. Neelam et al. [15] detected seven QTLs on rice chromosomes 2, 4, 5, 7, and 9 using a RIL population derived from a cross between RYT2668 (RFS resistant) and PR116 (RFS susceptible). Govindaiah et al. [16] detected two major QTLs on chromosomes 3 and 8 by genome-wide association mapping (GWAS). Huang et al. [17] mapped four RFS resistance QTLs on chromosomes 2, 9, 10, and 11 using BC1F2 and F_2_ populations derived from a hybrid of Xiushui47 and FS159. Overall, many RFS resistance QTLs have been identified. However, RFS resistance QTL mapping has been performed in only a few parental cultivars. Constructing multi-resistance QTL is considered to be an effective strategy to improve plant disease resistance. Fu et al. [18] identified a QTL on chromosome 12 by combining bulked segregant analysis with whole genome resequencing (BSA-seq) and a simple sequence repeat (SSR) marker. Therefore, there is an urgent need to screen resistant germplasm sources and identify more RFS resistance QTLs.

Currently, molecular markers are useful tools for mapping disease resistance genes in many plant species. Simple sequence repeat (SSR) markers have been widely used for gene mapping and marker-assisted selection in breeding because of their advantages over other markers, such as abundant polymorphism, wide distribution, and known map locations [19]. In recent years, some progress has been made in mapping QTL related to rice disease resistance using SSR markers. Ashkani et al. [20] constructed linkage maps and located QTLs using SSR markers for resistance to rice blast in 188 F_3_ populations derived from a cross between resistant and susceptible Malaysian rice varieties. Wu et al. [21] detected QTLs against rice stripe virus disease using SSR markers in 226 F_2_ populations derived from resistance hybridization. Channamallikarjuna et al. [22] detected rice sheath blight disease resistance and other agronomic trait QTL using 126 polymorphic SSR markers in 127 RILs from a cross between resistant Teqing and susceptible HP2216. Li et al. [11] identified seven QTLs conferring RFS resistance using SSR molecular markers.

In the present study, an SSR marker was used to identify large-effect QTLs for RFS resistance. A rice variety IR77298-14-1-2::IRGC117374-1 from the International Rice Research Institute was identified to be resistant to RFS. The F_2_ and F_4_ populations from crossing IR77298-14-1-2::IRGC117374-1 and susceptible line 9311 were developed for mapping RFS resistance QTLs. The objectives of this research were to screen a novel tightly linked RFS resistance molecular markers and resistance candidate genes and lay a foundation for the fine mapping and gene cloning of resistance-related genes in resistant variety IR77298-14-1-2::IRGC117374-1.

## 2. Materials and Methods

### 2.1. Plant Materials and Pathogen Culture

IR77298-14-1-2::IRGC117374-1 (male parent, P2), which was identified as resistant to RFS, was obtained from the International Rice Research Institute, Los Banos, Philippines, while the other parent, 9311 (female parent, P1), is a susceptible rice variety. The F_2_ and F_4_ populations of RILs were developed from a cross between IR77298-14-1-2::IRGC117374-1 and 9311. A total of 201 F_2_ individual plants and 131 F_4_ individual plants were selected for this study. These rice materials were grown in greenhouses at the experimental base of the Sichuan Academy of Agricultural Sciences, Chengdu, Sichuan, China.

The isolates of *U. virens* for inoculation (PX·D25, PJ1, and ZG99) were preserved in our laboratory in previous years. These isolates were cultured on potato sucrose agar (PSA) medium, and hyphae disks were placed in potato sucrose (PS) fluid medium. The cultures were incubated at 28 °C on a shaker at 140 rpm for 10 d. The hyphae and conidia of the three isolates were collected and mixed for inoculation.

### 2.2. Evaluation of Field Resistance to U. virens

The rice panicles of the parents and F_2_ and F_4_ segregating populations were injected with a conidial suspension concentration of 1 × 10^6^ mL^−1^ at seventh to eighth booting stage. After inoculation, all rice plants were grown with a 90–95% relative humidity at 25/30 °C (night/day) for 4 days [18]. Three weeks after inoculation, disease symptoms of rice panicles were observed, and the number of diseased grains per panicle and diseased panicles were investigated. Each panicle was scored based on a disease rating scale from 0 to 9. The disease index (DI) was used to evaluate RFS resistance in rice. The DI was calculated from the disease rating scale according to the method of Fu et al. [18]. The evaluation criteria of resistance and susceptibility were as follows: immune (I), disease index (DI) = 0; high resistance (HR), 0.0 < DI ≤ 5.0; disease resistance (R), 5.0 < DI ≤ 10.0; moderate resistance (MR), 10.0 < DI ≤ 20.0; moderate susceptibility (MS), 20.0 < DI ≤ 40.0; susceptibility (S), 40.0 < DI ≤ 60.0; and high susceptibility (HS), 60.0 < DI ≤ 100.0.

### 2.3. DNA Extraction and SSR Analysis

Genomic DNA was extracted from individual plants of the parents and F_2_ and F_4_ generation population individuals using the cetyltrimethylammonium bromide (CTAB) method [23]. DNA quality and integrity were checked using a 0.8% agarose gel. The extracted DNA was dissolved in 1× Tris EDTA (TE) buffer and stored at −20 °C for later use.

A total of 1271 SSR markers, which were obtained from the published National Rice Data Center (https://www.ricedata.cn/), were used to screen polymorphisms between the two parents, and the genotypes of the F_2_ and F_4_ populations were analyzed using markers with obvious polymorphism and clear and stable bands. For the SSR reaction, a 20 μL PCR mixture was used, containing 17 µL of Goldenstar T6 Super Mix (TsingKe, Beijing, China), 2 µL of 10 ng/µL of genomic DNA, and 1 µL of 0.5 µM forward and reverse primers. PCR amplification was performed according to the following conditions: initial denaturation at 95 °C for 5 min, followed by 35 cycles of denaturation at 95 °C for 30 s, annealing at 65 °C for 30 s, and extension at 72 °C for 30 s, and final extension at 72 °C for 5 min. The PCR products were separated using 6% acrylamide gel electrophoresis, and the bands were detected and read using the silver staining method [24].

### 2.4. Linkage Map Construction and QTL Analysis

The genotype bands of individuals of the F_2_ and F_4_ generation populations were analyzed. Alleles similar to the susceptible parent allele were scored as “A”; alleles similar to the resistant parent allele were scored as “B.” When both alleles were presented, it was scored as “H”, and when the allele was absent, it was scored as “-” [17]. The genotype data were used for linkage map construction by QTL IciMapping V4.0 software, with a minimum LOD value of 2.5 [25]. Phenotypic and genotypic data were combined to analyze the QTL. The identified QTLs were named according to the nomenclature reported by McCough and Doerge [26].

### 2.5. Candidate Gene Identification

QTL regions repeatedly detected in the F_2_ and F_4_ populations were considered as the candidate regions. The genes in the candidate regions were annotated using the Gene Ontology (GO), Non-redundant Protein Sequence (NR), and rice gene annotation databases (http://rice.uga.edu/index.shtml, accessed on 28 October 2024) to predict their functions as candidate genes related to RFS resistance.

### 2.6. Expression Analysis of Predicted Candidate Genes

To evaluate expression, we performed transcriptome analysis and reverse transcription quantitative PCR (RT-qPCR). Sample processing, sequencing, and data analysis methods for transcriptome analysis were conducted following the study of Fu et al. [27]. Briefly, total RNA was extracted from rice panicles using an RNA Extraction Kit (Aidlab Biotechnologies, Beijing, China). The obtained high-quality RNA was sent to Novogene, Co., Ltd. (Tianjin, China), for library construction, sequencing, and analysis.

The RT-qPCR was conducted using candidate genes with ubiquitin (*OsUBI*) as the internal reference. The primer sets for qRT-PCR were designed according to the individual gene sequences (Table 1). First, single-stranded cDNA reverse transcription was performed using the reverse transcription kit (Sangon Biotech, Co., Ltd., Shanghai, China). qPCR was then performed using Takara SYBR Green (Takara, Dalian, China). The 2^−∆∆CT^ method was used to calculate the relative gene expression levels [28].

## 3. Results

### 3.1. Phenotypic Analysis of QTL Populations Resistant to Rice False Smut

**Figure 1:**
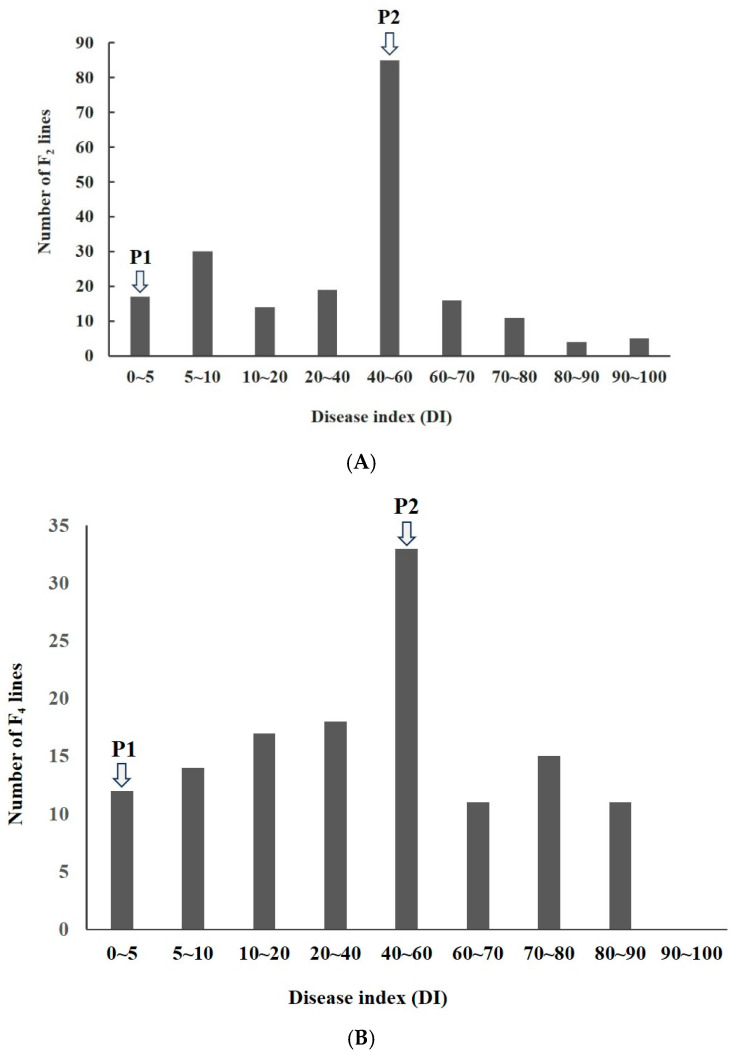
Frequency distribution of the rice false smut disease index (DI) of the F_2_ generation population (**A**) and F_4_ RILs (**B**) from an IR77298-14-1-2::IRGC117374-1 and 9311 cross.

The field resistance phenotype analysis of both parents and the F_2_ generation population showed that resistant parent IR77298-14-1-2::IRGC117374-1 (P2) did not have disease symptoms 21 d after inoculation, but susceptible parent 9311 (P1) produced many diseased grains after inoculation, with a DI above 50. The DIs of 201 individuals from the F_2_ generation population were between 0 and 100, showing a continuous phenotype distribution (Figure 1A). In addition, the disease resistance phenotype analysis of 131 F_4_ RILs showed that the DI had a continuous distribution, with DI ranging from 0 to 90 (Figure 1B), indicating that rice resistance to RFS had the genetic characteristics of a quantitative trait.

### 3.2. Screening of Polymorphic SSR Molecular Markers

In this study, 1271 SSR markers were tested using the genomic DNA template of parents 9311 and IR77298-14-1-2::IRGC117374-1, and SSR markers with polymorphism between the two parents were screened. We identified 119 SSR markers with good polymorphism that distinguished parental genotypes (P1 and P2) and heterozygous genotypes (F1), which were evenly distributed on 12 chromosomes.

### 3.3. Population Genotype Analysis and Genetic Linkage Map Construction

**Figure 2:**
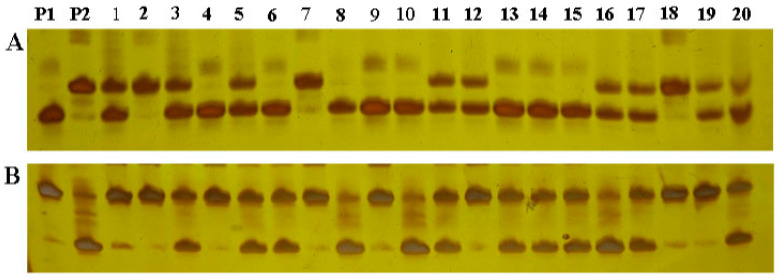
Segregation of SSR markers MR6959 (**A**) and MR5995 (**B**) in the F_2_ population.

**Figure 3:**
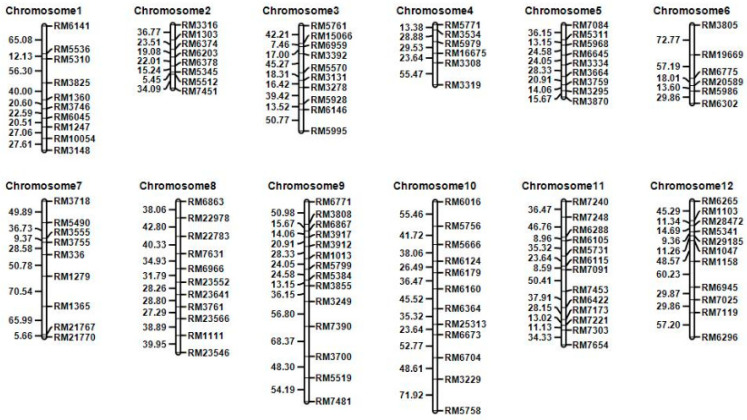
Linkage map of the F_2_ population was constructed by 119 SSR markers evenly distributed on 12 chromosomes.

The 201 individual plants from the F_2_ generation population and 131 F_4_ individual plants were genotyped using 119 markers that were polymorphic between parents. Figure 2 shows the genotype isolation of SSR markers MR6959 and MR5995 in some individuals from the population. QTL IciMapping4.0 software was used to analyze the marker genotypes and construct the genetic linkage map distributed on 12 chromosomes in rice according to the genotypes of the F_2_ and F_4_ populations. The F_2_ population covered a genetic distance of 2135.78 cM, and the average genetic map distance between molecular markers was 17.94 cM (Figure 3). The F_4_ population covered a genetic distance of 2640.12 cM, and the average genetic map distance between molecular markers was 22.18 cM (Supplementary Figure S1).

### 3.4. Screening of SSR Markers for RFS Resistance

SSR markers closely related to RFS resistance genes were identified by combining the phenotypic data (disease index) with the genotypic data of polymorphic SSR markers from individuals of the F_2_ and F_4_-isolated populations using QTL IciMapping4.0 software. We identified eight SSRs that were closely linked to the RFS resistance genes, which were located on chromosomes 1, 3, 5, and 12 (Table 2).

### 3.5. QTL Mapping of Rice False Smut Resistance Genes in Rice

**Figure 4:**
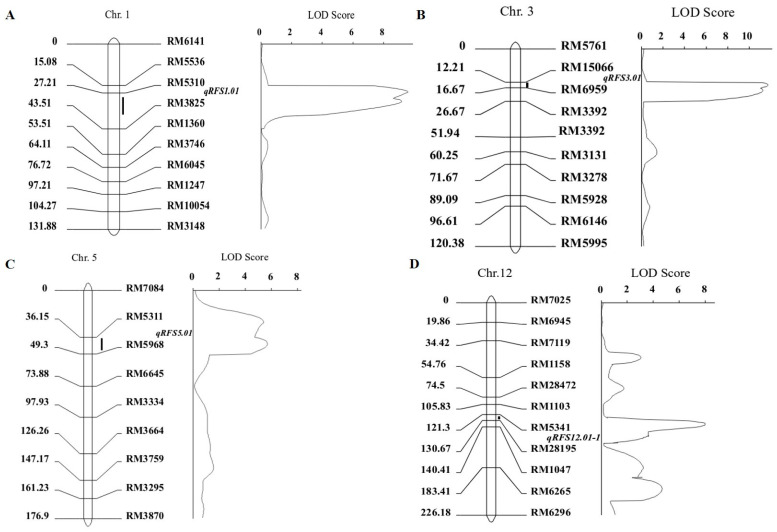
Four QTLs were identified on chromosomes 1 (**A**), 3 (**B**), 5 (**C**), and 12 (**D**) using the F_2_ population.

We combined genotypic and phenotypic data for QTL mapping by complete interval mapping using QTL IciMapping4.0 software. The LOD threshold was set to 2.5, and four QTLs were detected in the F_2_ and F_4_ populations, of which two intervals were repeatedly detected in both populations. Four resistance QTLs (*qRFS1.01*, *qRFS3.01*, *qRFS5.01*, and *qRFS12.01-1*) were detected in the molecular marker map constructed for the F_2_ population (Figure 4). Among them, *qRFS1.01* was located between RM5310 and RM3825, with an LOD value of 8.78, a phenotypic contribution rate of 6.63%, and an additive effect of 6.0443. The LOD value of *qRFS3.01* between RM15066 and RM6959 on chromosome 3 was 10.67, and this QTL had a phenotypic contribution rate of 37.73% and an additive effect of 21.72. *qRFS5.01* was located between RM5311 and RM5968 on chromosome 5, with an LOD value of 5.81, a phenotypic contribution rate of 9.74%, and an additive effect of 10.82. *qRFS12.01-1* was located between RM5341 and RM28195 on chromosome 12 and had an LOD value of 8.26, a phenotypic contribution of 29.74%, and an additive effect of 9.56. In addition, two resistance QTLs (*qRFS3.01* and *qRFS12.01-1*) were detected in the molecular marker map constructed for the F_4_ population (Supplementary Figure S2). Among them, *qRFS3.01* was located between RM15066 and RM6959 on chromosome 3 and showed an LOD value of 12.64, a phenotypic contribution of 45.73%, and an additive effect of 23.52. *qRFS12.01-1* was located between RM5341 and RM28195 on chromosome 12, with an LOD value of 2.76, a phenotypic contribution rate of 19.74%, and an additive effect of 9.56 (Table 3). The key QTLs for RFS resistance in rice were thus distributed on chromosomes on chromosomes 3 and 12 (*qRFS12.01-1*) as has been reported by Fu et al.) [18].

### 3.6. Analysis of Candidate Genes for Resistance to Rice False Smut

**Figure 5:**
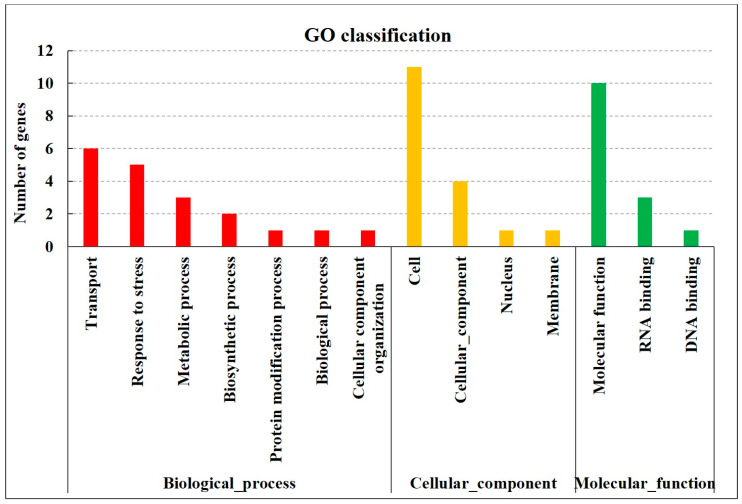
GO annotation of genes in candidate regions.

According to the results of QTL mapping, *qRFS3.01*, which controls RFS resistance in rice, was repeatedly detected on chromosome 3 in the F_2_ and F_4_ populations. To more accurately explore the genes related to RFS resistance, this repeat detection region was selected as the final candidate region. The physical distance of the repeat detection interval was 14.34–14.91 Mb, and the size of the region was 0.57 Mb, which was the region between SSR markers RM15066 and RM6959 (*qRFS3.01*) and contained 66 candidate genes, 49 of which were successfully annotated. These annotated genes fell into three main categories: biological processes, cellular components, and molecular functions (Figure 5).

The first category comprised genes involved in biological processes, such as transport (GO:0006810), response to stress (GO:0006950), metabolic processes (GO:0008152), biosynthetic process (GO:0009058), and cellular component organization (GO:0016043). Infection with *U. virens* is a biological stressor that can induce plant transcriptional regulatory factors, stress responses, and other biological processes to achieve pathogenicity. According to the bioinformatic function and expression site of rice gene annotation, two genes were predicted to be related to RFS resistance, namely, *LOC_Os03g25340* and *LOC_Os03g25430* (Supplementary Table S1).

The second category encoded genes with molecular functions, such as molecular function (GO:0003674), DNA binding (GO:0003677), kinase activity (GO:0016301), RNA binding (GO:0003723), and catalytic activity (GO:0003824). To resist pathogen invasion, rice has formed an extremely complex defense system in which some transcription factors, F-box proteins, and kinases are involved in disease resistance. Therefore, these genes, including *LOC_Os03g25304*, *LOC_Os03g25400*, *LOC_Os03g25480*, *LOC_Os03g25220*, *LOC_Os03g25250*, *LOC_Os03g25240,* and *LOC_Os03g25289*, were predicted to be involved in the regulation of RFS resistance (Supplementary Table S1).

The third category includes genes with cellular component, such as cell (GO:0005623), cellular component (GO:0005575), nucleus (GO:0005634), and membrane (GO:0005623). According to gene annotation, the genes related to the pathogenic function of *U. virens* were not found in this category.

In summary, three genes (*LOC_Os03g25240*, *LOC_Os03g25304*, and *LOC_Os03g25400*) were highly expressed in panicles, leaves, pistils, and anthers according to the rice genome annotation database (http://rice.uga.edu/index.shtml, accessed on 29 October 2024). Meanwhile, of these candidate genes, *LOC_Os03g25240* encodes an F-box domain-containing protein, namely, OsFBX89. *LOC_Os03g25304* encodes a Myb-like binding-domain-containing protein. *LOC_Os03g25400* encodes a kinase protein. These three genes were preliminarily predicted to be most correlated with RFS resistance.

### 3.7. Expression Analysis of Candidate Resistance Genes

The expression profiles of the three candidate genes between *U. virens* infection and mock inoculation in resistant and susceptible parents were analyzed by transcriptomics. The expression of the three genes was significantly upregulated in resistant parents (Table 1). In addition, the RT-qPCR experiment further confirmed that these three genes were significantly induced in resistant parents (Table 1). Therefore, we speculate that these three genes are closely related to RFS resistance.

## 4. Discussion

RFS is a serious fungal disease in rice-planting areas worldwide. Developing effective prevention and control strategies for RFS is an urgent task. The screening of RFS-resistant cultivars and the identification of genes/QTLs for RFS resistance may help to facilitate the control of this devastating disease. Previously, we demonstrated that rice variety IR77298-14-1-2::IRGC117374-1 was resistant to RFS [27]. In this study, multiple RFS resistance QTLs were identified on different chromosomes of rice variety IR77298-14-1-2::IRGC117374-1. SSR markers closely linked to these QTLs are of great value for developing excellent RFS-resistant varieties.

SSRs, which have lengths less than 200 bp, are widely distributed in different locations of the plant genome, and they are often used as molecular markers for genetic linkage analysis and QTL mapping [20,29]. In this study, SSR markers were used to identify QTLs conferring resistance to RFS. Polymorphism in SSR markers is the primary condition for gene mapping. In this experiment, 119 polymorphic primers were selected from 1271 SSR markers, accounting for 9.36% of the total number of markers, indicating that the polymorphism in SSR markers was low between resistant parent IR77298-14-1-2::IRGC117374-1 and susceptible parent 9311. The reason may be that the parents in the experiment have a close genetic relationship as they are both indica rice varieties, which would result in low marker polymorphism. The closer the genetic relationship between varieties, the lower the SSR polymorphism rate [11]. Therefore, parents with distant genetic relationships should be selected in future studies. The selection of indica and japonica hybridization results in a higher probability of polymorphism among markers [30].

In recent years, some researchers have studied the inheritance of RFS resistance. To date, more than 40 RFS resistance QTLs have been mapped to rice chromosomes in biparental populations generated by crossing resistant and susceptible varieties [15,17]. In this study, four QTLs were mapped to the F_2_ and F_4_ populations on chromosomes 1, 3, 5, and 12 based on the constructed genetic linkage map and resistance phenotype. Among these, two QTLs (*qRFS3.01* and *qRFS12.01-1*) were detected in both mapping populations. The *qRFS12.01-1* locus was located within the interval of the previously identified locus *qRFS12.01* [18]. Interestingly, QTL *qRFS3.01* is a novel resistance locus that has not been reported previously. *qRFS3.01* explained more than 36% of the phenotypic variance in RFS resistance QTLs, which was larger than that identified in previous studies. In our previous study, we already reported a contribution to the phenotypic variance of 28.74% for *qRFS12.01-1* [18]. Therefore, this QTL is another major RFS resistance locus. This QTL can significantly reduce the disease index and improve RFS resistance, and it is expected to be used in molecular-marker-assisted breeding for disease resistance.

To date, very few RFS resistance QTLs have been finely mapped, although resistance genes have been revealed [14,15]. In this study, we mapped an RFS resistance locus from resistant variety IR77298-14-1-2::IRGC117374-1, which helped us to identify more resistance genes. Based on gene prediction and annotation in the region of the *qRFS3.01* locus, several genes were predicted to be involved in RFS resistance. For instance, three genes, namely, *LOC_Os03g25240*, *LOC_Os03g25304*, and *LOC_Os03g25400*, encode one F-box domain-containing proteins, one Myb-like protein, and one kinase, respectively. Previous studies have shown that F-box proteins play important roles in plant responses to biotic stress caused by pathogen infection [31,32]. The F-box protein gene *OsDRF1* in rice upregulates the expression of defense-related genes (PR1a and Sar8.2b) to enhance disease resistance [33]. MYB transcription factors are among the most abundant and versatile transcription factors in plants, playing a positive regulatory role in the process of hypersensitivity response caused by pathogen infection [34,35]. In addition, kinase proteins mainly catalyze protein phosphorylation and play an important role in the rice stress response [36]. We confirmed that the expression of three genes was significantly upregulated in resistant rice by transcriptomics and RT-qPCR in this study. Therefore, these genes may be closely related to RFS resistance. However, further experiments should identify the functional characterization of the candidate genes by knocking out each of the candidate genes in the resistant parental genetic background, as well as by constructing overexpressing lines for each of the candidate genes in both the susceptible and the resistant parental genetic background.

## 5. Conclusions

Four QTLs for RFS resistance have been identified from IR77298-14-1-2::IRGC117374-1 by SSR marker mapping approaches. In particular, QTL *qRFS3.01* is a novel and valuable locus for RFS resistance. The *qRFS3.01*-linked SSR markers can be used in the marker-assisted selection of cultivars with RFS resistance. Some candidate genes associated with RFS resistance were predicted in this QTL region, but they need to be further validated by knocking out and constructing overexpressing lines for each of the candidate genes in both the susceptible and the resistant parental genetic background. These findings provide important information for isolating the genetic loci controlling RFS resistance and for developing rice cultivars with RFS resistance.

## Figures and Tables

**Table 1:** RNA-Seq and RT-qPCR validation of candidate genes.

Genes	Primers (5′–3′)	Log_2_FC (RNA-Seq)		Log_2_FC (RT-qPCR)	
		IR77298-14-1-2::IRGC117374-1	9311	IR77298-14-1-2::IRGC117374-1	9311
*LOC_Os03g25240*	F-GACGACCTGGTCGAGGAGATC	2.7293	0.2318	2.8109	0.5013
	R-GCCAAACAGAATGTGCGCTGCGC				
*LOC_Os03g25304*	F-CCGTCTTCCGCGGAGGCGGCGG	3.9273	0.3813	3.3146	0.2286
	R-TGCTGATGTCCTTCCACGACC				
*LOC_Os03g25400*	F-GAGAGGAACCTGCTGCGGTCGGC	2.3231	0.8712	1.8046	0.612
	R-AGCATGTTGAGCCACCAGCAT				

**Table 2:** List of SSR molecular markers linked to resistance genes of rice false smut.

Primer	Chromosome	Forward Primer	Reverse Primer
RM5310	1	GGGACCAAGACCTTTCCAATGC	GCGGAAGCAGGAGAATCGTAGC
RM3825	1	CCACTAGCAGATGATCACAGACG	GAGCACCTCATAAGGGTTTCAGC
RM15066	3	GCCGCAGTTGAGAGAACTCTTCC	GAGACGCGGATGACGAGACG
RM6959	3	GATTCCTATGGAGGATTGTTGC	AACTCCACCGGTGTTAAGAAGG
RM5311	5	CGTCTTGTCTAATCAGCTTAGGG	CACATCAAAGATATCGGGTTGG
RM5968	5	GGGTTACTGCACTACGGCATCG	GGTGGTGAATGGAAGGATCATGG
RM29185	12	CCTAGTTCAGCTCCTGCTTACC	CTCAGATGTAGGGAATGTTTGC
RM5341	12	CATCCGGAGGAAGTTTGAAAGAAGG	CAAGGGCAACCTCTTCCACTACGC

**Table 3:** Quantitative trait loci identified in the F_2_ and F_4_ populations for rice false smut resistance.

Traits	QTL	Chr.	Linkage Marker Flanking	LOD Value	Phenotypic Variance (%)	Additive Effect	Dom
F2	*qRFS1.01*	1	RM5310–RM3825	8.78	6.63	6.04	10.59
	*qRFS3.01*	3	RM15066–RM6959	10.67	37.73	21.72	10.91
	*qRFS5.01*	5	RM5311–RM5968	5.81	9.74	10.82	−1.79
	*qRFS12.01-1*	12	RM5341–RM28195	8.26	29.74	9.56	10.775
F_4_	*qRFS3.01*	3	RM15066–RM6959	12.64	45.73	23.52	10.91
	*qRFS12.01-1*	12	RM5341–RM28195	2.76	19.74	9.56	9.77

## Data Availability

Data are contained within the article.

## Supplementary Materials

The following supporting information can be downloaded at https://www.mdpi.com/article/10.3390/biom15020186/s1, Table S1: Candidate genes for resistance to rice false smut. Figure S1: Linkage map of the F_4_ population constructed using 119 SSR markers evenly distributed on 12 chromosomes. Figure S2: Two QTLs identified on chromosomes 3 (B) and 12 (D) using the F_4_ population.

## References

[1] Ashizawa T., Takahashi M., Arai M., Arie T. (2012). Rice false smut pathogen, *Ustilaginoidea virens*, invades through small gap at the apex of a rice spikelet before heading. J. Gen. Plant Pathol..

[2] Sun W., Fan J., Fang A., Li Y., Tariqjaveed M., Li D., Hu D., Wang W.M. (2020). *Ustilaginoidea virens*: Insights into an emerging rice pathogen. Annu. Rev. Phytopathol..

[3] Hu Z., Zheng L., Huang J., Zhou L., Liu C., Liu H. (2020). Ustiloxin A is produced early in experimental *Ustilaginoidea virens* infection and affects transcription in rice. Curr. Microbiol..

[4] Shan T.J., Sun W.B., Wang X.H., Fu X.X., Sun W.X., Zhou L.G. (2013). Purification of ustiloxins A and B from rice false smut balls by macroporous resins. Molecules.

[5] Chen X., Qiu J.H., Xiong M., Shu Y.Z., Huang S.W., Kou Y.J. (2019). Research progress of rice false smut. Chin. Rice.

[6] Zhang Y., Zhang K., Fang A., Han Y., Yang J., Xue M., Bao J., Hu D., Zhou B., Sun X. (2014). Specific adaptation of *Ustilaginoidea virens* in occupying host florets revealed by comparative and functional genomics. Nat. Commun..

[7] Zhou Y., Yu J., Pan X., Yu M., Du Y., Qi Z., Zhang R., Song T., Yin X., Liu Y. (2019). Characterization of propiconazole field-resistant isolates of *Ustilaginoidea virens*. Pestic. Biochem. Physiol..

[8] Xu J.L., Xue Q.Z., Luo L.J., Li Z.K. (2002). Preliminary report on quantitative trait loci mapping of false smut resistance using near-isogenic introgression lines in rice. Acta Agric. Zhejiangensis.

[9] Andargie M., Li L., Feng A., Zhu X., Li J. (2018). Mapping of the quantitative trait locus (QTL) conferring resistance to rice false smut disease. Curr. Plant Biol..

[10] Yang D.W., He N.Q., Huang F.H., Jin Y.D., Li S.P. (2023). The genetic mechanism of the immune response to the rice false smut (RFS) Fungus *Ustilaginoidea virens*. Plants.

[11] Li Y.S., Huang S.D., Yang J., Wang C.L. (2011). Analysis of Quantitative Trait Loci for Resistance to Rice False Smut. Acta Agron. Sin..

[12] Han Y., Li D., Yang J., Huang F., Sheng H., Sun W. (2020). Mapping quantitative trait loci for disease resistance to false smut of rice. Phytopathol. Res..

[13] Hiremath S.S., Bhatia D., Jain J., Hunjan M.S., Kaur R.K., Zaidi N.W., Singh U.S., Zhou B., Lore J.S. (2021). Identification of potential donors and QTLs for resistance to false smut in a subset of rice diversity panel. Eur. J. Plant Pathol..

[14] Qiu J., Lu F., Wang H., Xie J., Wang C., Liu Z., Meng S., Shi H., Shen X., Kou Y.J. (2020). A candidate gene for the determination of rice resistant to rice false smut. Mol. Breed..

[15] Neelam K., Kumar K., Kaur A., Kishore A., Kaur P., Babbar A., Kaur G., Kamboj I., Lore J.S., Vikal Y. (2022). High-resolution mapping of the quantitative trait locus (QTLs) conferring resistance to false smut disease in rice. J. Appl. Genet..

[16] Govindaiah M.D., Selvaraj R., Kadirimangalam S.R., Sundararajan A., Nachimuthu V.V., Swaminathan M., Ayyasamy R., Natarajan D., Ramasamy S., Dharmaraj D. (2022). Genetic dissection of false smut resistance in rice through genome wide association mapping. J. Phytopathol..

[17] Huang Y.F., Cai K.X., Zhang Z., Chai R.Y., Xie H.G., Shou J.Y., Fu J.R., Li G.L., Liu J.Y., Wu S.Q. (2023). Identification and fine-mapping of quantitative trait loci (QTL) conferring rice false smut resistance in rice. J. Genet. Genom..

[18] Fu R.T., Zhao L.Y., Chen C., Wang J., Lu D.H. (2024). Conjunctive analysis of BSA-Seq and SSR markers unveil the candidate genes for resistance to rice false smut. Biomolecules.

[19] Röder M.S., Korzun V., Wendehake K., Plaschke J., Tixier M.H., Leroy P., Ganal M.W. (1998). A microsatellite map of wheat. Genetics.

[20] Ashkani S., Rafi M.Y., Rahim H.A., Latif M.A. (2013). Mapping of the quantitative trait locus (QTL) conferring partial resistance to rice leaf blast disease. Biotechnol. Lett..

[21] Wu S.J., Zhong H., Zhou Y., Zuo H., Zhou L.H., Zhu J.Y., Ji C.-Q., Gu S.-L., Gu M.-H. (2009). Identification of QTLs for the resistance to rice stripe virus in the indica rice variety Dular. Euphytica.

[22] Channamallikarjuna V., Sonah H., Prasad M., Rao G.J.N., Chand S., Upreti H.C., Singh N.K., Sharma T.R. (2010). Identification of major quantitative trait loci qSBR11-1 for sheath blight resistance in rice. Mol. Breed..

[23] Murray M.G., Thompson W.F. (1980). Rapid isolation of high molecular weight plant DNA. Nucleic Acids Res..

[24] Lim S.E., Sa J.K., Lee J.K. (2021). Bulk segregant analysis identifies SSR markers associated with leaf-and seed-related traits in Perilla crop (*Perilla frutescens* L.). Genes Genom..

[25] Meng L., Li H.H., Zhang L.Y., Wang J.K. (2015). QTL IciMapping: Integrated software for genetic linkage map construction and quantitative trait locus mapping in biparental populations. Crop J..

[26] McCough S.R., Doerge R.W. (1995). QTL mapping in rice. Trends Genet..

[27] Fu R.T., Chen C., Wang J., Zhao L.Y., Chen X.J., Lu D.H. (2022). Evaluation and screening of rice germplasm resources resistant to rice false smut. J. South. Agric..

[28] Livak K.J., Schmittgen T.D. (2001). Analysis of relative gene expression data using real time quantitative PCR and the 2^−∆∆CT^ Method. Methods.

[29] Rahman M.A., Thomson M.J., De Ocampo M., Egdane J.A., Salam M.A., Shah-E-Alam M., Ismail A.M. (2019). Assessing trait contribution and mapping novel QTL for salinity tolerance using the Bangladeshi rice landrace capsule. Rice.

[30] Chen Q.Q., Zhang Y.S. (2009). Distorted segregation and construction of molecular linkage map of SSR markers in Indica rice. Mol. Plant Breed..

[31] Jia Q., Xiao Z.X., Wong F.L., Sun S., Liang K.J., Lam H.M. (2017). Genome-wide analyses of the soybean F-box gene family in response to salt stress. Int. J. Mol. Sci..

[32] Stone S.L. (2014). The role of ubiquitin and the 26S proteasome in plant abiotic stress signaling. Front. Plant Sci..

[33] Cao Y., Yang Y., Zhang H., Li D.Y., Zheng Z., Song F.M. (2008). Overexpression of a rice defense related F-box protein gene *OsDRF1* in tobacco improves disease resistance through potentiation of defense gene expression. Physiol. Plant..

[34] Ambawat S., Sharma P., Yadav N.R. (2013). MYB transcription factor genes as regulators for plant responses: An overview. Physiol. Mol. Biol. Plants.

[35] Raffaele S., Rivas S., Roby D. (2006). An essential role for salicylic acid in *AtMYB30*-mediated control of the hypersensitive cell death program in *Arabidopsis*. FEBS Lett..

[36] Zhang M.M., Zhang S.Q. (2022). Mitogen-activated protein kinase cascades in plant signaling. J. Integr. Plant Biol..

