# Supplementary figures and images for: Identification of Quantitative Trait Loci and Analysis of Novel Candidate Genes for Resistance to False Smut of Rice Based on SSR Molecular Markers
Source: Biomolecules. 2025 Jan 28;15(2):186. doi: 10.3390/biom15020186 (PMC11852790; doi:10.3390/biom15020186)

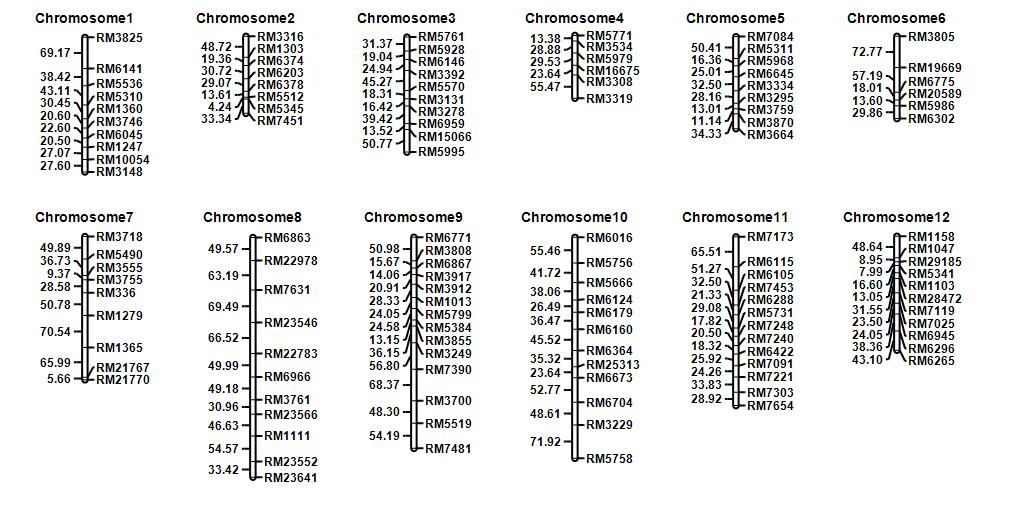

Supplement: Supplementary file 1 [file biomolecules-15-00186-s001.zip › biomolecules-3409065-supplementary/Supplementary file/Supplementary Figure S1.jpg]

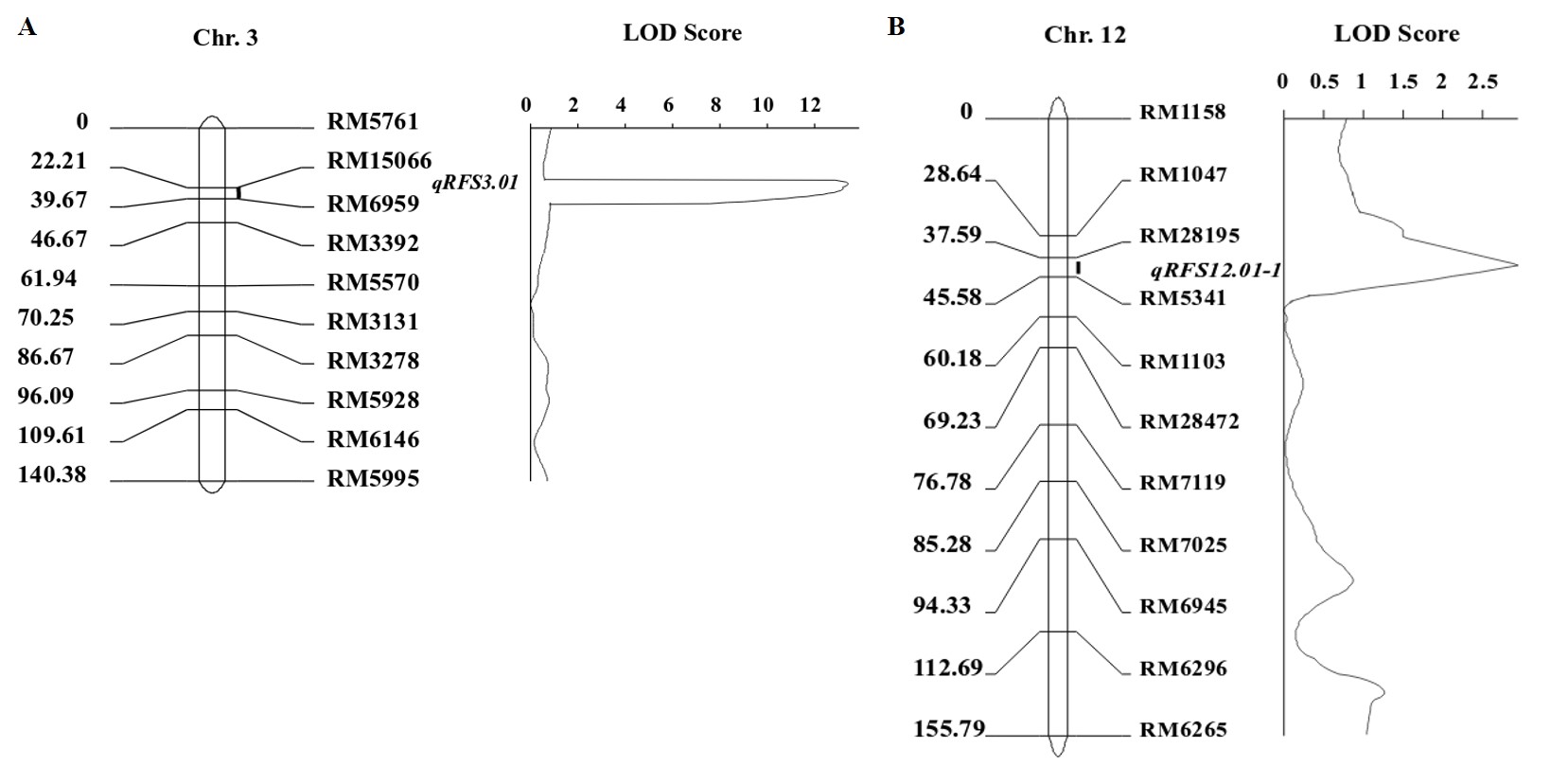

Supplement: Supplementary file 1 [file biomolecules-15-00186-s001.zip › biomolecules-3409065-supplementary/Supplementary file/Supplementary Figure S2.jpg]
